# Allostatic Load Assessment for Early Detection of Stress in the Workplace in Egypt

**DOI:** 10.3889/oamjms.2016.066

**Published:** 2016-06-16

**Authors:** Ola Sayed Ali, Nadia Badawy, Sanaa Rizk, Hend Gomaa, Mai Sabry Saleh

**Affiliations:** 1 *Department of Biochemistry, Faculty of Pharmacy, Al-Azhar University, Cairo, Egypt*; 2 *Department of Environmental and Occupational Medicine, National Research Center, Cairo, Egypt*

**Keywords:** Allostatic load, stress, job satisfaction;occupational stress, chronic diseases

## Abstract

**AIM:**

Workplace stress is hazardous for its harmful impact on employees’ health and organizational productivity. The aim of the study is to apply the Allostatic Load Index (ALI) which is a multi-component measure for health risk assessment and early detection of stress among workers in Egypt.

**METHODS:**

Sixty-two working adults randomly selected from two different working environments in Egypt were included in the study. Participants completed a self-reported questionnaire for socio-demographic and work variables. Andrews and Withey test for Job Satisfaction was filled and 3 ml blood samples were collected. Markers assessed for Allostatic Load were serum cortisol, c-reactive protein, dehydroepiandrosterone-sulphate, total thyroxine, total cholesterol, triglycerides, low-density lipoprotein, high-density lipoprotein, total cholesterol to high-density lipoprotein ratio, systolic and diastolic blood pressures, waist to hip ratio and body mass index. The risk quartile method was used for calculation of ALI. ALI value of four or more indicates high Allostatic Load.

**RESULTS:**

Job satisfaction scale defined about a quarter of the study population (24%) to be dissatisfied with Allostatic Load of 2.4 as the mean value. Population percentage with ALI ≥4 reached 12.9% with 100% of them females. A significant association was found between Allostatic Load of primary mediators and age, the presence of chronic diseases, place of work and female gender.

**CONCLUSION:**

Female gender and the old age of the Egyptian workforce under study are at higher risk of chronic diseases. Using an alternative way -for example, the cut-point method- instead of the risk quartiles for dichotomization of markers used in ALI calculation could be more precise for early detection of stress among healthy individuals.

## Introduction

Workplace stress is an issue of growing interest in the field of Occupational health. It is defined by the Canadian Centre for Occupational Health and Safety [1] as the harmful physical and emotional responses that can result from conflicts between job demands on the employee and the amount of control an employee has over meeting these demands. Unfortunately, the decreased ability of workers in developing countries to recover from job stressors due to lack of awareness could seriously contribute to undesirable health conditions [2] beside the negative impact on workplace productivity and profit.

Allostatic load (AL) model [3], is a powerful way to assess physiological dysregulations in the state of prolonged secretion of stress hormones [4], stress hormones in conjunction with pro- and anti-inflammatory cytokines represent the AL biomarkers referred to as the primary mediators (primary stage). Secondary outcomes (secondary stage), include metabolic (e.g., insulin, glucose, total cholesterol, high-density lipoprotein, cholesterol, triglycerides, visceral fat depositing), cardiovascular (e.g., systolic and diastolic blood pressure), and immune (e.g. fibrinogen, C-reactive protein) parameters that reach sub-clinical levels. The final stage of AL progression is allostatic overload; in which physiological dysregulations lead to disordered, diseased, and deceased endpoints referred to as tertiary outcomes. The AL model proposes that by measuring the multi-systemic interactions among primary mediators and effects, in conjunction to sub-clinically relevant biomarkers representing secondary outcomes, biomedical advances can be made in the detection of individuals at high risk of tertiary outcomes [5].

The aim of the present study is to apply the multiple biochemical measures of AL for early detection of workers at high risk of debilitating drawbacks of workplace stress in Egypt. Working environment related variables including job satisfaction and some socio-demographic factors will be studied as predisposing factors to workplace stress.

## Subjects and Methods

A sample of 62 Egyptian working adults (18 males and 44 females) completed the study. The mean age of the study sample participants was 40 years ranging from 20 to 59 years. Thirty-five of them work in the different departments of the Faculty of Pharmacy (Girls), Al-Azhar University, Egypt and 27 works at the pediatric oncology outpatient clinic, National Cancer Institute (NCI), Egypt. Ethical comity at the National Research Center approved the study design and procedure was done under its guidance.

All participants were asked to fill a self-reported questionnaire including socio-demographic data (gender, age, social status, residence and history of chronic diseases) and some work related variable encompassing presence or absence of a second work, number of networking hours per day, total number of working years and job nature (worker or employee). Andrews and Withey Job Satisfaction Scale [6] was included in the questionnaire.

A 3 ml whole blood sample was taken from the study participants for biochemical analysis of AL biomarkers. The blood was collected in a plain red top venipuncture tube without additives or anti-coagulants for serum. Blood was allowed to clot for serum samples. Centrifugation of the specimen was done to separate the serum from the cells. Samples were refrigerated at -4 degrees Celsius for few days before analysis.

For determination of AL, biochemical markers measured as primary mediators were serum cortisol and dehydroepiandrosterone-sulphate (DHEA-s), c-reactive protein (CRP) and total thyroxine (tT4). Secondary outcome biomarkers were total cholesterol (TC), HDL-cholesterol, LDL-cholesterol and triglycerides (TG).

Serum cortisol and tT4 were assayed by an enzyme-linked immunosorbent assay (ELISA) technique using Immunospect kit (CA). DHEA-s and CRP were estimated in serum using ELISA kit manufactured by DRG diagnostics, Germany. TC, TG, and HDL-cholesterol in plasma were determined by a kinetic method using the Bio-diagnostic kit (Egypt) according to the methods described by Allain et al. [7], Fassati and Principe [8] and Burstein [9], respectively. LDL was estimated with reference to measured values of TC, HDL, and TG according to the Modified Friedewald Formula (MFF): LDL-C (mg/dl) = Non-HDL-C × 90% - TG × 10% [10].

Systolic blood pressure (SBP), diastolic blood pressure (DBP) and anthropometric measures; body mass index (BMI) and waist-to-hip ratio (WHR) were also measured to be included in the AL score. SBP and DBP were calculated as the average of two seated blood pressure readings taken about one minute apart, using a mercury sphygmomanometer [11]. WHR was calculated based on waist circumference (measured at its narrowest point between the ribs and iliac crest) and hip circumference (measured at the maximal buttocks) [12] and BMI was calculate from measured data as weight in kilograms divided by height in squared meters [11]. Total cholesterol to high-density lipoprotein cholesterol (TC/HDL) was calculated for determination of Allostatic load index (ALI).

All parameters chosen for determination of ALI were chosen according to the literature [13-14]. For each biomarker, the high-risk threshold was calculated and each participant was assigned a point for each biomarker that was beyond the threshold. The high-risk threshold was defined as below the 25^th^ percentile for DHEA-s and HDL and above the 75^th^ percentile for all other markers according to each measurement’s distribution within the population under study (Table 1). The points were summed to generate the ALI, with a range from 0 to 13. According to similar research, an ALI of four or greater was used to define a high AL [15-16].

**Table 1:** Criteria for contribution from individual biological parameters for ALI calculation

Highest quartile
SBP (≥ 150 mm Hg)
DBP (≥ 100 mm Hg)
WHR (≥ 1.5)
TC/HDL (≥ 10.4)
Serum cortisol (≥15.85 .g/dl)
CRP (≥7.6 mg/l)
Total thyroxin (≥12.4 .g/dl)
BMI (≥38.1)
TG (≥231 mg/dl)
TC (≥253 mg/dl)
LDL (≥171 mg/dl)
Lowest quartile
HDL cholesterol (≤ 49.8 mg/dl)
DHEA-S (≤ 1.02 .g/ml)

Descriptive statistics were represented as mean and standard deviation for studied variables and measures using the statistical package for social sciences, version 16 for windows (SPSS Inc., USA). Pearson Chi-square and student t-test were used for comparing data.

## Results

Descriptive data of study population as represented in Table 2 shows that percentage of females exceeds males in the study with 71% and 29%, respectively. Most of the study population are married (69.4%), don’t have a second job (81%), live in urban residence (82%), work for more than five hours per day (76%) and work as employees (71%).

**Table 2:** Distribution of the study variables

Study Variables	Frequency (%)
Gender	
Male	18 (29%)
Female	44 (71%)
Age	
<40	27 (44%)
≥40	35 (56%)
Work Place	
Al-Azhar	35 (56.5%)
NCI	27 (43.5%)
Social Status	
Married	43 (69.4%)
Others	19 (30.6%)
Other Job	
Present	11 (18%)
Absent	50 (81%)
Chronic Diseases	
Present	24 (38.7%)
Absent	38 (61.3%)
Residence	
Urban	51 (82%)
Rural	8 (13%)
Daily Working Hours	
≤ 5	13 (21%)
> 5	47 (76%)
Working Years	
≤ 10	24 (38.7%)
> 10	35 (56.5%)
Job Nature	
Employee	44 (71%)
Worker	18 (29%)
Job Satisfaction	
<20	47 (76%)
≥20	15 (24%)

Nearly neither quarter of the population (24%) under study shows to be dissatisfied or neither satisfied nor dissatisfied with their job.

Table 3 shows the means with standard deviations together with minimum and maximum values of the different biomedical measures under study, ALI for all measures, ALI for primary mediators and job satisfaction scale. Mean value for cortisol (11.4 µg/dl) lies nearly at the middle of the normal range (5-23 µg/dl) as well as cholesterol (183 mg/dl) which has a normal range of 150-225 mg/dl.

**Table 3:** Descriptive statistics of biomedical measures in the study

Variable (N)	Mean ± SD	Minimum	Maximum
Cortisol (62)	11.4 ± 3.7	4.3	19.7
DHEA-s (62)	0.8 ± 0.6	0.2	3.5
CRP (62)	4.9 ± 3.3	0.5	10.1
T4 (62)	9.8 ± 2.7	4.7	15.1
TC (62)	183 ± 66	140	284
TG (62)	122 ± 52	48	292
HDL (62)	61 ± 28	22	99
LDL (62)	96 ± 54	1	228
TC/HDL (62)	3.5 ± 2.2	1.2	13.5
SBP (62)	122.8 ± 14.6	90	170
DBP (62)	81 ± 8.3	60	113
BMI (62)	31.5 ± 6.6	18	48
WHR (62)	0.88 ± 0.15	0.6	1.85
Job Satisfaction	15.4 ± 4.4	8	25
ALI (62)	2.4 ± 1.2	0	5
ALI of Primary Mediators(62)	1.44 ± 0.92	0	4

As for DHEA-s (0.8 µg/mlL) the mean value is very near to the lower normal (0.56 µg/ml) while CRP (4.9 mg/l), tT4 (9.8 µg/dl), TG (122 mg/dl), HDL (61 mg/dl) and LDL (96 mg/dl) means greatly approach the highest values within their normal ranges; 0.068-8.2 mg/l, 5-13 µg/dl, 40-140 mg/dl for women and 60-165 mg/dl for men, 30-70 mg/dl for men and 35-85 mg/dl for women and >120 mg/dl, respectively. Mean values for systolic (122.8) and diastolic (81) blood pressures are normal while BMI mean (31.5) exceeds obesity threshold (30). Total ALI (2.4) showed to be higher than ALI for primary mediators (1.44).

Mean values of ALI didn’t show to be significantly different with respect to work related and socio-demographic variables considered in the study (Table 4). On the other hand gender (p = 0.036), age (p = 0.013), workplace (p = 0.000) and presence of chronic diseases (p = 0.014) significantly affected ALI of primary mediators as shown in Table 4. Females (1.6), higher age group (1.7), participants working at Faculty of Pharmacy Al-Azhar University (1.8) and participants with chronic diseases (1.8) showed higher values of ALI for primary mediators than males (1.1), lower age group (1.1), participants working at NCI (0.9) and participants not suffering from chronic diseases (1.2), respectively.

**Table 4:** Comparing means for Allostatic load according to studied socio-demographic and work-related variables of the study population

Study Variables	Allostatic Load Index	ALI of Primary Mediators
Gender		
Male	2.0 ± 0.8	1.1 ± 0.8*
Female	2.5 ± 1.3	1.6 ± 0.9
Age		
<40	2.1 ± 1.3	1.1 ± 0.9*
≥40	2.5 ± 1.2	1.7 ± 0.9
Work Place		
Al-Azhar	2.5 ± 1.1	1.8 ± 0.9**
NCI	2.1 ± 1.3	0.9 ± 0.68
Social Status		
Married	2.3 ± 1.2	1.4 ± 0.9
Others	2.5 ± 1.2	1.5 ± 0.9
Other Job		
Present	2.4 ± 1.1	1.5 ± 0.8
Absent	2.3 ± 1.2	1.4 ± 1.0
Chronic Diseases		
Present	2.7 ± 1.2	1.8 ± 1.0*
Absent	2.1 ± 1.2	1.2 ± 0.8
Residence		
Urban	2.4 ± 1.2	1.5 ± 0.9
Rural	2.1 ± 0.8	1.4 ± 0.9
Daily Working Hours		
≤ 5	2.5 ± 1.1	1.7 ± 1.0
> 5	2.3 ± 1.2	1.4 ± 0.9
Working Years		
≤ 10	2.3 ± 1.5	1.2 ± 1.0
> 10	2.4 ± 1.0	1.6 ± 0.9
Job Nature		
Employee	2.4 ± 1.3	1.5 ± 1.0
Worker	2.3 ± 1.1	1.4 ± 0.7
Job Satisfaction		
<20	2.4 ± 1.2	1.4 ± 0.85
≥20	2.1 ± 1. 4	1.57 ± 1.13

* p ≤ 0.05;

** p ≤ 0.01.

No significant difference was detected between the population sample showing high ALI (≥ 4) and those with low ALI (< 4) regarding the different variables tested in the study except for gender where 100% of the population with high ALI was found to be females (Table 5).

**Table 5:** Relation between Allostatic Load Index (ALI) and study variables

Variables (N)		ALI		Chi- square	P-value
		< 4 N = 54	≥ 4 N = 8		
Gender (62)	Male	33%	0%	3.76	0.015*
	Female	66%	100%		
Age (62)	<40	44%	37.5%	0.137	0.710
	≥40	56%	62.5%		
Social status (62)	Married	70%	62.5%	0.203	0.675
	others	30%	37.5%		
Chronic Diseases (62)	Present	37%	50%	0.494	0.487
	Absent	63%	50%		
Residence (59)	Urban	81%	87.5%	1.246	0.140
	Rural	15%	0%		
Work Place (62)	Al-Azhar	56%	62.5%	0.137	0.710
	NCI	44%	37.5%		
Working Years (59)	<10	37%	50%	0.892	0.350
	≥10	59%	37.5%		
Working Hours (60)	≤ 5	22%	12.5%	0.254	0.599
	> 5	76%	75%		
Other Job (61)	Present	18.5%	12.5%	0.191	0.650
	Absent	79.5%	87.5%		
Job Nature (62)	Employee	70%	75%	0.072	0.785
	Worker	30%	25%		
Job Satisfaction	<20	76%	75%	0.003	1.00
	≥20	24%	25%		

* p≤0.05.

## Discussion

Upon assessment of AL for our randomly selected sample of working adults in Egypt, ALI was found to be 2.4 as the mean value. Fortunately, this value of AL score is lower than values obtained by the similar cross-sectional studies on the workforce like those done in Canada (2.69) [17], Germany (3.15) [18] and China (3.69-4.54) [19]. At the time where only one study in Netherland showed a lower range of means of AL (1.7-7-2.03) for a population sample of males working as telecom managers [20] compared to our results.

However, according to results of the assessed markers, risk quartile thresholds of TC (253 mg/dl), TG (231 mg/dl), LDL (171 mg/dl), TC/HDL (≥ 10.4), DBP (≥ 100 mmHg), WHR (≥ 1.5) and BMI (38.1) highly jumped over the corresponding threshold ranges detected in similar studies as reported by Mauss and his colleagues [21] and even exceeded the normal ranges. As reported in their review, ranges of threshold values showed to be 177.9–249.0 mg/dl, 101.5–141.75 mg/dl, 116.0–137.3 mg/dl, 3.71, 71.2–95.0 mm Hg, 0.83–0.97, 25.2–28.5, respectively which is too much lower. The threshold for DHEA-s according to lowest risk quartile was ≤1.02 μg/ml that is also lower than the corresponding thresholds reported (13.3–51.5 μg/dl).

Such results reflect a seriously bad general health condition for our study population compared to others and render the mean value for AL deceiving and reflecting a false indication of the good state of health. Moreover, in our consideration, if the same data obtained from the present study were recomputed but using the cut-point method [22] for calculation of AL, a higher mean value for AL will be detected. Using the quartile method allowed a high percentage of the population to skip being rated on the ALI despite their absolute values for certain measures exceeded the normal range which could be indication of serious load (see for BMI as an example where the population with the range 30-38 were rated with 0 for AL since the highest quartile threshold is 38.1). This could be regarded as a disadvantage of the risk quartile method for dichotomization of AL measures, and invites more research studying the best indicative way for ALI calculation.

Work-related variables under study including job satisfaction didn’t show significant influence on AL score in agreement with findings of Johansson and his colleagues [23]. No associations were detected, except for a place of work where population working at Al-Azhar University suffered from significantly higher (p<0.01) AL of primary mediators (1.8) compared to those working at the NCI (0.9) at p<0.01. AL of primary mediators reflects the allostatic state phase of the AL sequence [24] which is the state preceding AL and allostatic overload. The importance of detection of primary mediators lies on the fact that they represent nonclinical measures that provide additional warning signs of bad health above and beyond that shown by standard clinical measures as stated by Goldman et al. [25]. Similarly, ALI mean values for Al-Azhar University (2.5) showed to be higher than that of NCI workers mean (2.1) but, non-significantly, which ensures the worth health conditions of the former over the later.

Job satisfaction is a crucial indicator for measuring work wellbeing [26]. About quarter of the study population (24%) appeared to score more than or equals to twenty with a mean value of 15.4 for the whole population. This value for job satisfaction could be indicating a trend of dissatisfaction or at least carelessness of the issue. No association was detected between ALI and job satisfaction scores as demonstrated by the present work in contrast with the study hypothesis which assumed there would be a direct association between the two measures due to the reported effect of job satisfaction on physical and psychological wellbeing [27].

Many studies reported a direct association between high AL and increased age [14, 28-29]. Age appeared to affect AL prominently in our results where AL was 2.5 for the higher age group (≥ 40 years) than the lower age group (2.1), also AL for primary mediators was significantly higher (p = 0.013) in the older group (1.7) than the other group (1.1). The adverse effect of age on AL could be attributed to the fact that AL measures the cumulative biological risk normally increased with age as stated by Crimmins et al., [30].

Although both males (29%) and females (71%) were represented in the study population yet, all the percentage of the sample (12.9%) that showed high AL (ALI ≥ 4) were females. Moreover, means of both ALI and that of primary mediators showed to be higher in females (2.5 and 1.6, respectively) compared to males (2.0 and 1.1, respectively) with p = 0.036 for AL of primary mediators. These results are in contrast with Schnorpfeil et al., (2003) and Li (2007) [18, 28] who recorded a positive association between AL and the male gender and may indicate sever life conditions and health state for women in Egypt.

The presence of chronic diseases also showed to be associated with high AL (2.7) compared to population free from chronic diseases (2.1). Significantly higher AL of primary mediators (1.8 vs. 1.1, p = 0.014) for a population with chronic diseases was also detected. These results are in agreement with the reported significantly increased AL with decreased physical health for Latino day workers in the USA as stated by De Castro et al., [31], the decreased self-rated health recorded by Naswall [32] in Sweden and the increased physical complaints as detected by Juster and Lupien [17] in Canada.

In conclusion, despite diversity among prior research on AL, regarding inclusion of biomarkers, method of calculation of AL, type of study and population under examination which makes it difficult to compare results of different studies, yet a growing confidence in the great worth of using the multi-component AL measure as predictor for health risk assessment is emphasized. According to our study, results signified bad health conditions for our working adults, especially women.

Work-related variables like effort-reward imbalance, work safety, job control, job demands and burnout that proved direct association with AL in previous studies made on various communities are needed to be investigated in Egypt and other developing countries. Socio-economical status as reported by De Castro et al. together with age and gender as discussed earlier also represent important predictors of stress and health deterioration.

Re-launching similar studies over larger population for screening purposes in Egypt and trying other methods for calculation of risk thresholds are highly recommended putting in mind that the present work could be regarded as a pilot exploratory cross-sectional study.
